# Long-term diagnosis-specific sickness absence, disability pension, and healthcare use in 1305 young adult childhood cancer survivors and in 6430 references; a Swedish ten-year prospective cohort study

**DOI:** 10.1371/journal.pone.0275343

**Published:** 2022-09-30

**Authors:** Fredrik Baecklund, Kristina Alexanderson, Lingjing Chen

**Affiliations:** 1 Department of Microbiology, Tumor and Cell Biology, Karolinska Institutet, Stockholm, Sweden; 2 Pediatric Oncology Unit, Karolinska University Hospital, Stockholm, Sweden; 3 Division of Insurance Medicine, Department of Clinical Neuroscience, Karolinska Institutet, Stockholm, Sweden; Flinders University, AUSTRALIA

## Abstract

**Background:**

Childhood cancer survivors (CCS) are at high risk of chronic health conditions. We aimed to explore young adult CCS’ and matched references’ future diagnoses-specific healthcare use, sickness absence (SA), and disability pension (DP).

**Methods:**

We performed a prospective cohort study with microdata from seven nationwide Swedish registers. We included 1305 young adult CCS born 1983–1988 and living in Sweden in 2008 and 6430 matched references and followed them for ten years (2009–2018) regarding mean annual specialized outpatient visits, inpatient days, and SA (spells >14 days) and/or DP (SADP) days, overall and by eight diagnostic groups. Risk factors for >90 SADP days in 2018 were explored as odds ratios (OR) with 95% confidence intervals (CI) by adjusted logistic regression.

**Results:**

Approximately 80% of CCS and 90% of references did not have SADP in the ten-year follow-up. Mean SADP days/year was higher among CCS (40–50 days/year), particularly in CNS tumor survivors (76–83 days/year), compared to references (12–18 days/year). Most SADP days were DP days. CCS had more mean outpatient visits (1.6–1.8 visits/year) and inpatient days (0.8–1.7 days/year) than references (0.8–1.2 visits/year and 0.6–0.75 days/year, respectively). The main healthcare use and SADP diagnoses were neoplasms and psychiatric disorders among all CCS, along with nervous system and endocrine conditions among CNS tumor survivors. The risk of SADP >90 days in 2018 was higher among female compared to male CCS (OR = 2.34, 95% CI 1.67–3.32), those with elementary schooling compared to high school/university education (OR = 6.52, 95% CI 4.49–9.49), and survivors of CNS tumors compared to other malignancies (OR hematological versus CNS = 2.88, 95% CI 1.95–4.28; OR hematological versus non-CNS solid tumors = 0.71, 95% CI 0.45–1.09).

**Conclusions:**

Most CCS did not have SADP as young adults; nevertheless, their risk of SADP was higher than among matched references. CNS tumor survivors were at particularly high risk of SADP.

## Background

The five-year survival after childhood cancer diagnosis has improved substantially in the last decades and has reached 80% in high-resource countries [1]. However, childhood cancer survivors (CCS) are at higher risk of chronic health conditions compared to individuals without childhood cancer [2–6]. Studies have shown an overall higher risk of hospitalization [7–12], outpatient visits [12, 13], and prevalence of chronic health conditions (all grades) (as high as 77% of all CCS with an average of 3–4 diagnoses/CCS [2, 14]) among CCS compared to among references without childhood cancers. Although the prevalence of chronic health conditions, hospitalization, and outpatient visits is higher among young adult CCS than references, their burden in terms of affected work capacity might vary largely.

Particularly, a few studies have shown that CCS have a higher risk of social security benefit uptake such as disability pension (DP) compared to references [15–19]. For example, in a Norwegian birth cohort of 1965–1985 followed until 2009, CCS had a 5.6 times higher risk of DP (indicating long-term disability) and 17.9 times higher risk of attendance benefit (indicating up to one year of sickness absence (SA)) compared to references [17]. The most common DP diagnoses were neoplasms, and endocrine and CNS related conditions, while for attendance benefits the most common reasons were neoplasms, injury and poisoning, and diseases of the sense organs [17]. Higher risks of DP and attendance benefits [17], and other social benefits [15, 16, 19] were observed among women compared to men [17, 19], and survivors of central nervous system (CNS) tumors compared to other childhood cancers [15–17, 19]. While these studies indicate a higher risk of SA/DP among CCS compared to references, if and how this risk vary over follow-up time was not investigated. Information on the diagnoses behind the social benefits were only included in one [17] of these abovementioned studies [15–19] and this is thus not well studied. Nevertheless, patients and their families, healthcare providers, employers, insurance companies, and society at large have questions about the possible future life situation among young adult CCS, e.g., regarding need of healthcare and of SA/DP, for instance when planning for preventive measures, for education and for work. Such questions need proper answers, based on high-quality data with long-term follow-up.

Thus, the aim of this study was to investigate future diagnoses-specific specialized healthcare use, sickness absence, and disability pension among young adult female and male CCS and matched references.

## Methods

We conducted a ten-year prospective cohort study of young adult CCS and their population-based matched references. The study was based on anonymized micro-data from seven Swedish nationwide government-administered registers. Data was linked at individual level by use of the unique personal identity number given all residents of Sweden [20].

### Data sources and type of data

Registers held by Statistics Sweden: The Swedish Total Population Register keeps record of all residents in Sweden, for data on sex and date of birth [21]. The Multi Generation Register [22] for data on peoples parents. The Longitudinal Integration Database for Health Insurance and Labor Market Studies (LISA) [23] for data on socioeconomics, including educational attainment, type of living area, birth country, and migration.

Registers held by the Swedish National Board of Health and Welfare: The Swedish Cancer Register [24] holds data on newly diagnosed cancers since 1958 with a coverage of about 96%. Registered data include diagnosis date and cancer type as International Classification of Disease version 7 (ICD-7) [25]. The Swedish National Patient Register [26, 27] contains information on dates and diagnoses (ICD) for all inpatient care episodes since 1987 and specialized outpatient physician visits since 2001. The Cause of Death Register [28] registers date of deaths in Sweden.

Register held by the Swedish Social Insurance Agency: The Micro Data for Analyses of Social Insurance (MiDAS) [29] is an administrative register with information on all SA spells >14 days and all DP, including start- and end-date, extent (full- or part-time), and diagnosis (as ICD-10 codes [30] based on the medical certificate provided by the patient’s treating physician).

### Study cohorts

The CCS and reference cohorts were identified among all individuals born 1983–1988, who were alive and lived in Sweden in December 2008, which was defined as baseline (thus, aged 20 to 25 years at baseline). The CCS cohort included all individuals with a cancer diagnosis registered in the Swedish Cancer Register before aged 18 years who were alive ≥5 years after the date of the cancer diagnosis. A reference cohort was created by randomly selecting five individuals for each CCS among the remaining individuals of the relevant ages and living in Sweden and who were free of childhood cancer, individually matched to the CCS by sex, birth year, birth country (Sweden or not), and parents’ highest educational level when the index person was 15 years of age. In total, 1305 CCS and 6430 references formed the study cohort at baseline.

*Childhood cancer diagnoses* were categorized into three main categories using ICD-7 codes: hematological malignancies, including lymphoma (200–202, 205–206) and leukemia (203–204, 207); primary central nervous system (CNS) tumor (193.0–193.2); and non-CNS solid malignancies. The non-CNS solid malignancies included neuroblastoma (193.3–193.9), retinoblastoma (192, 199.1), renal cancer (180), bone sarcoma (196), soft tissue sarcoma (197), germ cell tumors (175, 178), and others. *Age* at first cancer diagnosis was categorized as 0–4, 5–9, 10–14, and 15–17 years. Sociodemographic variables were categorized as follows: *birth country* (Sweden or other), *migration* during follow-up (yes or no), *educational attainment* (elementary [≤9 years], high school [10–12 years], or university/college [>12 years]), and *type of living area* (big city, urban, or rural).

All people in Sweden aged ≥16 years with income from work or unemployment benefits can be granted SA benefits if their work capacity is reduced due to disease or injury [31]. Because sick-pay for the first 14 days of a SA spell is provided by the employer, and thus not registered in MiDAS, we only included SA spells >14 days. All residents in Sweden aged 19–64 years with long-term or permanent work incapacity due to disease or injury can be granted DP. When aged 19–29, temporary DP can also be granted if morbidity necessitates more time to complete education. SA benefits amount to about 80%; DP to about 64% of lost income, both up to a certain level. If having had no income before granted DP a minimum sum/month is paid. Both SA and DP can be granted at four levels of ordinary work hours (25%, 50%, 75%, or 100%)—this means that an individual can have both part-time SA and DP at the same time.

### Outcome measures

The main outcome was annual mean SA and DP net days among the young adult CCS and references. We calculated net days of SA and DP, e.g., two gross days of 50% absence constitute one SADP net day (short as SADP day below). In analyses of potential risk factors of SADP among CCS, SADP days was categorized into ≤90 and >90 days in 2018. The choice of cut-off at 90 days had several reasons; we wanted to catch the long-term absences, not those based on flues, non-serious fractures, etc. this cut-off is often used in studies of SADP, and in the Swedish system people are assessed for the possibility to do other job tasks when a SA spell reaches 90 days.

We also calculated the annual mean number of specialized outpatient physician visits and the annual mean number of inpatient healthcare days among the young adult CCS and references.

The SA and DP diagnoses as well as the diagnoses for inpatient and specialized outpatient healthcare were categorized into the following eight groups: “neoplasms” (ICD-10 codes C00-C97, D00-D48), “endocrine (E00-E90), “psychiatric” (F00-F99), “nervous system” (G00-G99), “musculoskeletal” (M00-M99), “pregnancy related” (O00-O99), “injury” (S00-T98), and “other” (remaining ICD-10 codes and missing).

### Statistical analyses

The frequency of sociodemographic and clinical characteristics for the study population at baseline were calculated as absolute numbers and proportions. The annual proportions of CCS and of references having had any inpatient visits or specialized outpatient visits, any SA, any DP, or any SA and/or DP were calculated for the ten-year follow up.

The annual mean number of inpatient days and outpatient healthcare visits, mean annual SA, DP, and SADP days, respectively, were calculated for each of the ten years 2009–2018 for CCS overall and for references, as well as stratified by the three main types of childhood cancer (hematological, CNS, non-CNS solid malignancies) among the CCS, and also stratified by sex. These measures were also calculated using those having had these types of visits/days the respective year as numerators.

The percentage of the eight diagnostic groups underlying inpatient care, outpatient visits, SA and DP, respectively, were calculated for each year of follow-up among CCS and references. In secondary analyses, these calculations were also performed among those CCS and references that had inpatient days, outpatient visits, and SA and DP, respectively.

In all above measures, CCS and references who died or emigrated during follow-up were included in the analyses until the year of event but were censored from the numerator the following years. In total, 52 CCS (3.7%) and 31 references (0.5%) died, and 35 CCS (2.5%) and 236 references (3.5%) emigrated during follow-up.

The associations between categories of exposure variables and having >90 SADP days in 2018 were estimated among CCS and references separately, using a crude univariate and an adjusted multivariate logistic regression model. For both CCS and references, the adjusted model included baseline values of the covariates index persons’ age, and highest educational level, parents’ highest educational level, type of living area, and birth country. In the analyses of CCS, the models were further adjusted for cancer type (hematological, CNS, non-CNS solid malignancies) and age at childhood cancer diagnosis.

All statistical analyses were performed using the software R version 4.0.4 [32].

The study was conducted in accordance with the World Medical Association Declaration of Helsinki [33]. Participant consent is generally not required in large register-based studies in the Nordic countries [34] and was waived by the Regional Ethical Review Board in Stockholm, Sweden, who approved of the project. All data were anonymized by the administrative authorities before delivered to us.

## Results

Distribution of baseline sociodemographic characteristics among CCS and references, and childhood cancer characteristics among CCS, are presented in Table 1. Age at baseline was evenly distributed between 20–25 years. In 2008, 82.5% of CCS and 87.9% of references had at least some high school or university/college education. Higher proportions of CCS than among references had DP at inclusion. The proportion of CCS and references with SA was 4.9% and 5.0%, with DP was 12.6% and 2.8%, and with SADP was 17.1% and 7.7%, respectively. A larger proportion of women than men had SADP at baseline, both among CCS (19.6% versus 14.8%) and references (8.9% versus 6.6%).

**Table 1:** Distribution of sociodemographic characteristics at baseline (2008) among childhood cancer survivors and matched references, and childhood cancer characteristics among childhood cancer survivors; among all and stratified by sex.

	Childhood cancer survivors			References		
	Total	Women	Men	Total	Women	Men
	N (%)	n (%)	n (%)	N (%)	n (%)	n (%)
**Total**	1305	628	677	6430	3085	3345
**Sex**						
Women	628 (48.1)	628 (100)		3085 (48.0)	3085 (100.0)	
Men	677 (51.9)		677 (100)	3345 (52.0)		3345 (100)
**Age**						
20	206 (15.8)	93 (14.8)	113 (16.7)	1025 (15.9)	463 (15.0)	562 (16.8)
21	199 (15.2)	87 (13.9)	112 (16.5)	990 (15.4)	433 (14.0)	557 (16.7)
22	233 (17.9)	117 (18.6)	116 (17.1)	1147 (17.8)	573 (18.6)	574 (17.2)
23	229 (17.5)	121 (19.3)	108 (16.0)	1132 (17.6)	596 (19.3)	536 (16.0)
24	215 (16.5)	95 (15.1)	120 (17.7)	1056 (16.4)	466 (15.1)	590 (17.6)
25	223 (17.1)	115 (18.3)	108 (16.0)	1080 (16.8)	554 (18.0)	526 (15.7)
**Country of birth**						
Sweden	1248 (95.6)	597 (95.1)	651 (96.2)	6154 (95.7)	2935 (95.1)	3219 (96.2)
Other	57 (4.4)	31 (4.9)	26 (3.8)	276 (4.3)	150 (4.9)	126 (3.8)
**Educational level**						
Elementary	228 (17.5)	115 (18.3)	113 (16.7)	778 (12.1)	324 (10.5)	454 (13.6)
High school or university/college	1077 (82.5)	513 (81.7)	564 (83.3)	5652 (87.9)	2761 (89.5)	2891 (86.4)
**Parent’s highest educational level**						
Elementary	98 (7.5)	50 (8.0)	48 (7.1)	509 (7.9)	259 (8.4)	250 (7.5)
High school	590 (45.2)	272 (43.3)	318 (47.0)	2867 (44.6)	1341 (43.5)	1526 (45.6)
University/college	617 (47.3)	306 (48.7)	311 (45.9)	3054 (47.5)	1485 (48.1)	1569 (46.9)
**Type of living area**						
Big city	453 (34.7)	233 (37.1)	220 (32.5)	2268 (35.3)	1134 (36.8)	1134 (33.9)
Urban	576 (44.1)	268 (42.7)	308 (45.5)	2766 (43.0)	1315 (42.6)	1451 (43.4)
Rural	276 (21.1)	127 (20.2)	149 (22.0)	1396 (21.7)	636 (20.6)	760 (22.7)
**Any sickness absence in spell >14 days**	64 (4.9)	30 (4.8)	34 (5.0)	320 (5.0)	196 (6.4)	124 (3.7)
**Any disability pension**	164 (12.6)	97 (15.4)	67 (9.9)	181 (2.8)	82 (2.7)	99 (3.0)
**Sickness absence and/or disability pension**	223 (17.1)	123 (19.6)	100 (14.8)	495 (7.7)	275 (8.9)	220 (6.6)
**Age at first cancer diagnosis**						
0–4	432 (33.1)	214 (34.1)	218 (32.2)			
5–9	313 (24.0)	129 (20.5)	184 (27.2)			
10–14	350 (26.8)	160 (25.5)	190 (28.1)			
15–17	210 (16.1)	125 (19.9)	85 (12.6)			
**Childhood cancer diagnosis**						
***Hematological***	484 (37.1%)	220 (35.0%)	264 (39.0%)			
Lymphoma	203 (15.6)	83 (13.2)	120 (17.7)			
Leukemia	281 (21.5)	137 (21.8)	144 (21.3)			
***CNS***	341 (26.1)	166 (26.4)	175 (25.8)			
***Non-CNS solid tumor***	480 (36.7%)	242 (38.5%)	238 (35.1%)			
Neuroblastoma	36 (2.8)	18 (2.9)	18 (2.7)			
Retinoblastoma	55 (4.2)	23 (3.7)	32 (4.7)			
Renal	63 (4.8)	31 (4.9)	32 (4.7)			
Bone	50 (3.8)	22 (3.5)	28 (4.1)			
Soft tissue sarcomas	49 (3.8)	21 (3.3)	28 (4.1)			
Germ cell tumors	69 (5.3)	38 (6.1)	31 (4.6)			
Others	158 (12.1)	89 (14.2)	69 (10.2)			

Note: CNS = central nervous system, N = number of individuals.

**Fig 1:**
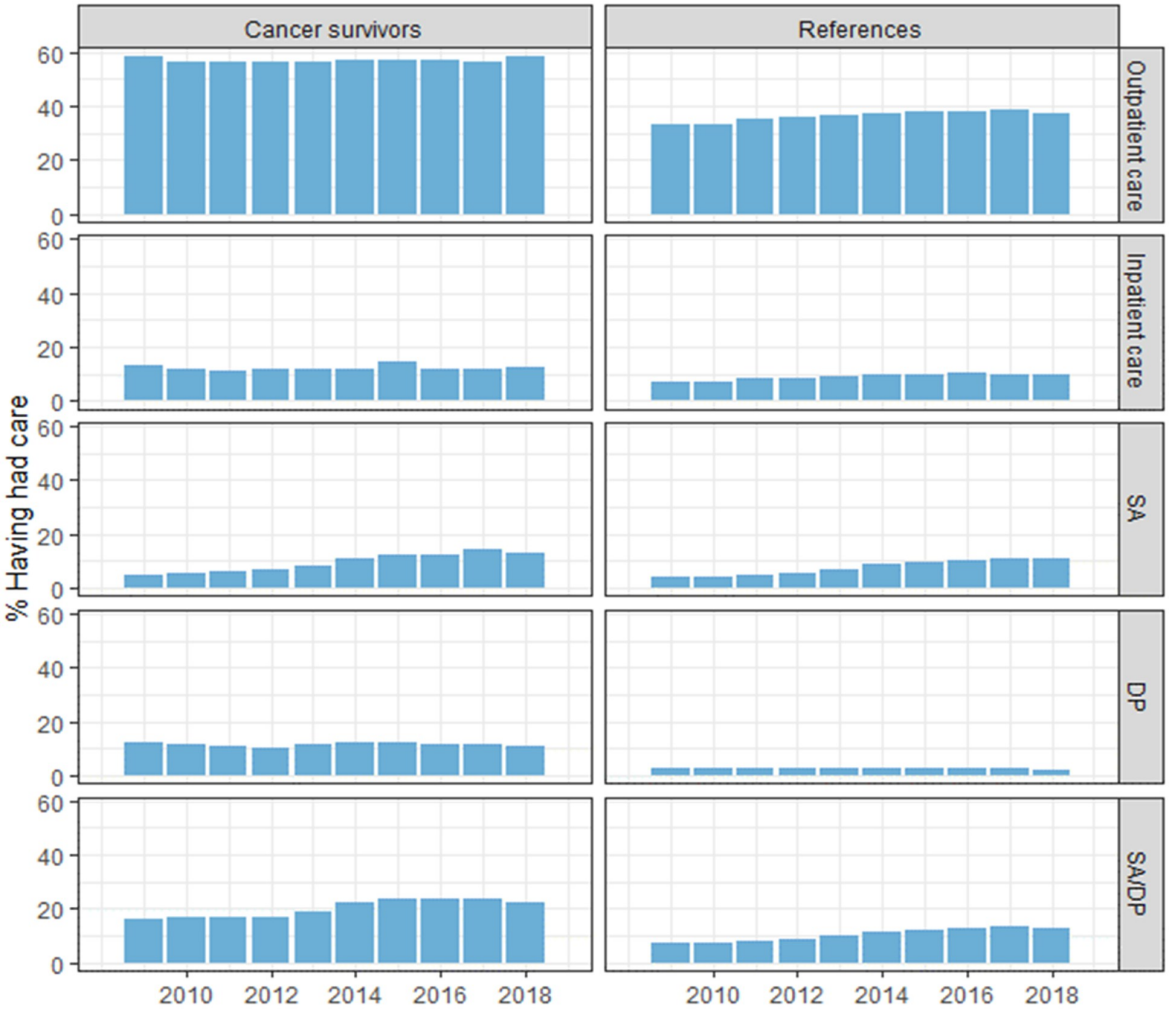
The annual proportions (%) of childhood cancer survivors and matched references who each of the years 2009–2018 had specialized outpatient care, inpatient care, sickness absence (SA), disability pension (DP), and sickness absence and/or disability pension (SA/DP), respectively.

During the ten-year follow-up period, the proportion with any specialized outpatient healthcare visits varied between 50–60% among CCS and 30–40% among references (Fig 1). The proportions who had any inpatient healthcare were slightly higher among CCS (range 11.0–14.4%) than references (range 7.2–10.4%), with the greatest difference in 2009–2012. A slightly larger proportion of CCS than references had SA (CCS range 4.7–14.6%, references range 4.7–11.3%), while the proportions who had DP differed more (CCS range 11.1–12.7%, references range 2.2–2.8%). The proportions with SADP differed annually between CCS (range 16.7–24.7%) and references (range 7.4–13.3%) during follow-up.

Overall, the annual mean number of diagnosis-specific outpatient healthcare visits was higher among CCS (range 1.7–2.3 visits/year) than among references (range 0.8–1.2 visits/year), and among women than among men (S1 Fig in S1 File). The number of outpatient visits/year were about the same in the three main categories of childhood cancers (approximately 2–3.5 visits/year among women and 1–2 visits/year among men). The most common diagnoses category for outpatient visits was “other”. Among CCS, visits due to neoplasms and psychiatric conditions were relatively common. In addition, a notable proportion of CNS tumor survivors had visits due to endocrine and nervous system disorders.

The annual mean number of diagnosis-specific inpatient days varied considerably between follow-up years among CCS while they were more stable among references (S2 Fig in S1 File). Overall, the mean number of inpatient days was higher among CCS (approximately 0.5–3 days/year) than references (approximately 0.5–1 day/year), and among women than men. The proportions of main diagnoses for inpatient care varied between years. Psychiatric conditions were among the most common reasons for inpatient care, especially among female CCS, and among male and female references. Neoplasms was a more common reason among CCS than references. Additional reasons for inpatient care were nervous system disorders among survivors of CNS tumors, and pregnancy-related conditions among women.

**Fig 2:**
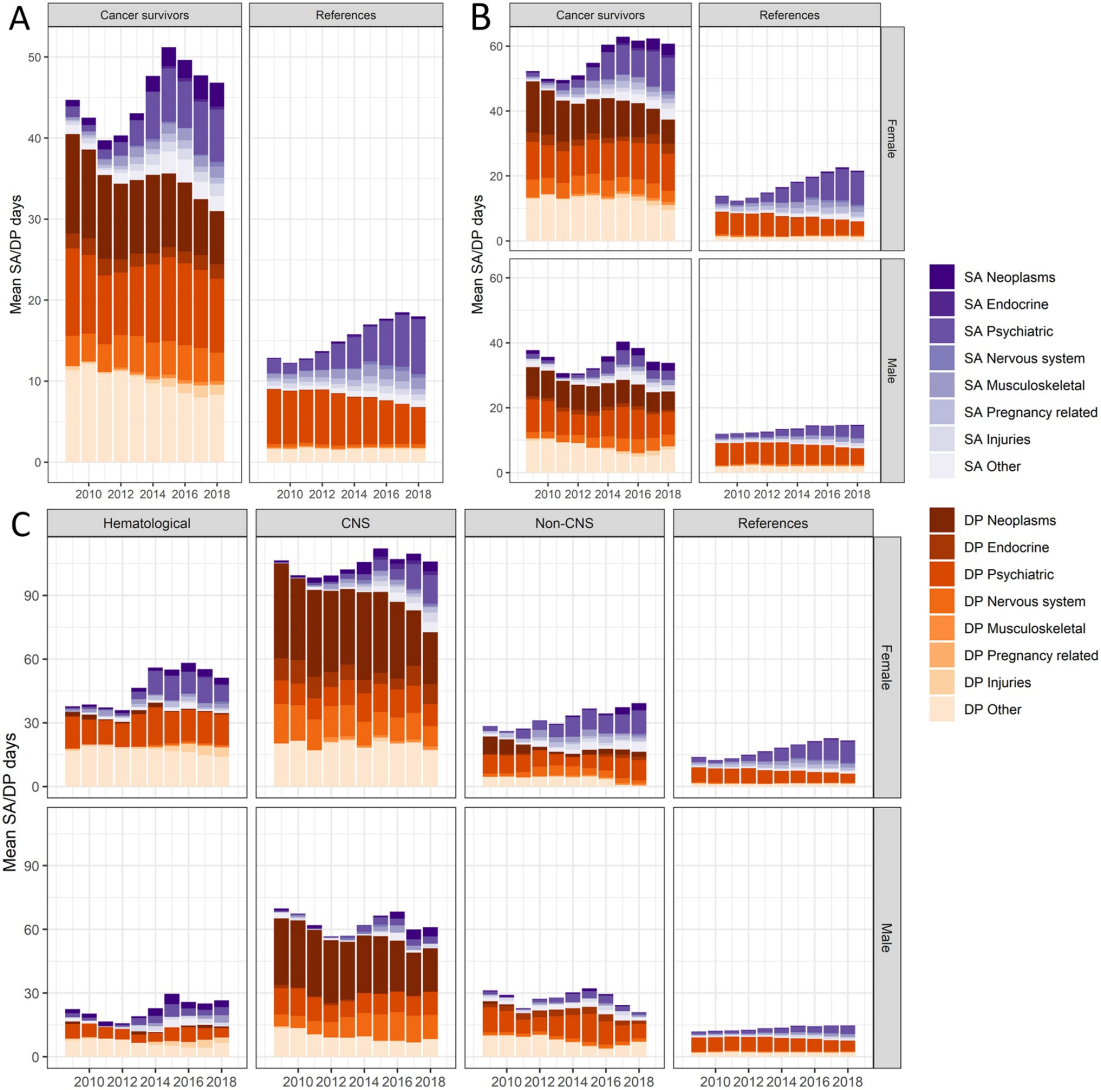
The annual mean number of sickness absence (SA) and disability pension (DP) net days in general and by type of SA and DP diagnoses in 2009 through 2018 among young adult childhood cancer survivors and their matched references (A); stratified by sex (B); and by sex and main types of childhood cancer (C).

The annual mean number of diagnosis-specific SADP days among CCS and references are presented in Fig 2. SADP was more common among CCS than references, and among women than men. The highest annual mean number of SADP days was observed among survivors of CNS tumors (women around 100 days/year, men around 60 days/year). DP was the dominating reason for SADP days among all CNS tumor survivors, female hematological malignancies survivors, and male non-CNS solid malignancies survivors, throughout follow-up; in all other subgroups SA days gradually increased to become as common as DP at the end of follow-up. Among CNS tumor survivors, the most common SADP diagnoses were neoplasms, psychiatric, nervous system, and endocrine (women only). Among survivors of hematological and non-CNS solid malignancies, and among references, psychiatric diagnoses were the most common SADP diagnoses. The number of study subjects available each follow-up year are provided in S1 Table in S1 File.

When limiting the explorative analyses to those CCS who had SADP the respective year of follow-up (S3 Fig in S1 File), the annual mean number of SADP days varied between 200–300 days among CNS tumor survivors, 150–250 days among survivors of hematological malignancies, and between 120–250 days among survivors of non-CNS solid malignancies. Among the references, the annual mean SADP days varied between 150–200 days.

After adjustments, the following factors were associated with a higher risk of SADP >90 days in 2018 among both CCS and references (Table 2): female compared to male sex (CCS OR 2.34, 95% CI 1.67–3.32; references OR 1.85, 95% CI 1.49–2.31) and having elementary schooling compared to higher educational level (high school and university/college; CCS OR 6.52, 95% CI 4.49–9.49; references OR 6.45, 95% CI 5.10–8.16). Among CCS, those diagnosed with CNS tumor were at higher risk of SADP >90 days, compared to those with hematological malignancy (OR 2.88, 95% CI 1.95–4.28). There was no statistical difference in the risk of SADP >90 days between survivors of hematological and non-CNS solid malignancies (OR 0.71, 95% CI 0.45–1.09). Among women, the adjusted risk of SADP >90 days in 2018 was higher among survivors of non-CNS solid malignancies (OR 1.60, 95% CI 1.03–2.41), hematological malignancies (OR 2.59, 95% CI 1.74–3.80), and CNS tumors (OR 7.36, 95% CI 5.06–10.7) compared to references. Among men, the adjusted risk of SADP >90 days in 2018 was higher among survivors of hematological malignancies (OR 1.87, 95 CI 1.11–3.01) and CNS tumors (OR 5.73, 95% CI 3.70–8.73), but not among survivors of non-CNS solid malignancies (OR 1.60, 95% CI 0.89–2.72), compared to references.

**Table 2:** Crude and adjusted odds ratio (OR) and 95% confidence intervals (CI) for sickness absence and/or disability pension >90 days in 2018 among childhood cancer survivors and among references, respectively.

	Childhood cancer survivors			References		
	N (%)	Crude	Adjusted	N (%)	Crude	Adjusted
		*OR (95% CI)*	*OR (95% CI)*		*OR (95% CI)*	*OR (95% CI)*
**Sex**						
Men	631 (52)	*Ref*	*Ref*	3211 (52)	*Ref*	*Ref*
Women	591 (48)	2.05 (1.51–2.81)	**2.34 (1.67–3.32)**	2956 (48)	1.64 (1.33–2.03)	**1.85 (1.49–2.31)**
**Educational level**						
High school or university/collage	1019 (83)	*Ref*	*Ref*	5424 (88)	*Ref*	*Ref*
Elementary	203 (17)	5.84 (4.16–8.20)	**6.52 (4.49–9.49)**	743 (12)	5.88 (4.71–7.34)	**6.45 (5.10–8.16)**
**Parents’ educational level**						
University/college	573 (47)	*Ref*	*Ref*	2904 (47)	*Ref*	*Ref*
High school	558 (46)	0.97 (0.70–1.33)	0.78 (0.54–1.12)	2774 (45)	1.30 (1.04–1.62)	0.98 (0.77–1.24)
Elementary	91 (7)	1.44 (0.82–2.43)	1.08 (0.56–1.99)	489 (8)	1.69 (1.17–2.39)	0.98 (0.66–1.43)
**Type of living area**						
Big cities	424 (35)	*Ref*	*Ref*	2152 (35)	*Ref*	*Ref*
Urban	541 (44)	1.26 (0.89–1.80)	1.21 (0.82–1.80)	2659 (43)	0.88 (0.69–1.12)	0.86 (0.68–1.11)
Rural	257 (21)	1.47 (0.97–2.21)	1.38 (0.87–2.20)	1356 (22)	1.07 (0.82–1.41)	1.05 (0.79–1.40)
**Country of birth**						
Sweden	1170 (96)	*Ref*	*Ref*	5908 (96)	*Ref*	*Ref*
Other	52 (4)	0.91 (0.39–1.86)	0.65 (0.25–1.55)	259 (4)	1.14 (0.67–1.81)	0.82 (0.47–1.36)
**Age at baseline 2008**						
20–22	600 (49)	*Ref*	*Ref*	3043 (49)	*Ref*	*Ref*
23–25	622 (51)	1.20 (0.89–1.63)	1.39 (0.99–1.96)	3124 (51)	1.23 (1.00–1.52)	**1.32 (1.07–1.64)**
**Age at cancer diagnosis**						
0–4	402 (33)	*Ref*	*Ref*			
5–9	298 (24)	1.12 (0.75–1.65)	1.10 (0.70–1.71)			
10–14	326 (27)	0.85 (0.57–1.27)	0.92 (0.59–1.44)			
15–17	196 (16)	0.84 (0.52–1.33)	0.85 (0.49–1.44)			
**Childhood cancer diagnosis**						
Hematological	454 (37)	*Ref*	*Ref*			
CNS tumor	321 (26)	2.75 (1.92–3.96)	**2.88 (1.95–4.28)**			
Non-CNS solid tumor	447 (37)	0.74 (0.49–1.11)	0.71 (0.45–1.09)			

Note: Adjusted model includes all variables in the table. Values of educational levels, type of living area, and age are those at baseline (2008). Statistically significant adjusted associations are highlighted with bold text. CNS = central nervous system, N = number of individuals.

## Discussion

In this ten-year prospective register-based cohort study, we explored the future diagnosis-specific healthcare use and SADP among young adult CCS and matched references. We found that most of the CCS had no SA nor DP in any of the ten years, even if the proportion who did was larger among CCS than among the references—especially regarding DP. Furthermore, the mean number of SADP days/year during the 10-year follow-up was below 50 days among CCS and below 20 days among references.

We found higher annual mean specialized outpatient visits, inpatient care days, and SADP days among CCS than among references, and among women than among men. While there was no clear difference in healthcare use between the three main categories of CCS, survivors of CNS tumors had two to three times more SADP days/year than survivors of other cancers, the majority of which were due to DP. The most frequent SADP diagnoses were neoplasms, psychiatric, nervous system, and endocrine disorders. Women, people with lower educational attainment, and CNS tumor survivors were at higher risk of >90 SADP days in the last year of follow-up (2018).

Studies have reported that the prevalence of chronic health conditions is higher among CCS than among references without childhood cancer [2–6]. However, having a diagnosis does not necessarily translate into having functional limitations, nor that such limitations lead to work incapacity in relation to the type of job the person has/seeks. Most people with different types of diagnoses are not on SA or DP due to them [35]. This seems also to be the case in our CCS cohort, given that each year of follow-up more than 50% had an outpatient visit with an accompanying diagnosis, while only about 20% had SADP days. The same applied to the references, where the corresponding numbers were more than 30% and about 15%, respectively.

Although the annual mean number of specialized outpatient visits was higher among CCS than among references, they were few in both groups: in the range of 2–3 visits/person/year among female CCS and 1–1.5 visits/person/year among male CCS, about twice the numbers observed among references. The most common cause for visit was “other”, indicating a large variety of reasons for outpatient visits. Of the seven more specific diagnoses categories, neoplasms and psychiatric disorders dominated. Additionally, some CNS tumor survivors had visits due to endocrine and nervous system disorders. Our findings are in line with previous studies that also found a higher risk of outpatient visits [12, 13] due to secondary malignancy (neoplasms), endocrine, and neurological conditions [13] among CCS than among references without such medical history.

We also observed a somewhat larger proportion having had inpatient care among CCS than among references (11–14.4% versus 7.2–10.4%). The mean number of inpatient days were also slightly higher among CCS than references, although they were few in both groups (0.5–3 versus 0.5–1 days/patient/year). Similar to the results regarding outpatient visits, the most common reasons for inpatient care were neoplasms (especially the first years of follow-up), psychiatric, and nervous system disorders. These findings are in line with previous studies showing a higher risk of hospitalization among CCS compared to references [7–12]. Neoplasms and conditions affecting the nervous system were among the most frequent conditions behind the hospitalizations in most [7, 10, 11] but not all [9] previous studies. Taken together, the results regarding healthcare use and underlying diagnoses from our and previous studies may indicate a higher burden of disease among CCS than among references, mainly due to neoplasms, psychiatric, endocrine, and neurological conditions. However, the reason for an outpatient visit could span from a planned check-up without active disease to conditions limiting the patient in his/her everyday life, qualities that are not reflected in the present or previous studies. Inpatient care may capture more severe health conditions than outpatient visits do, since the underlying diagnoses require an intervention (at least admittance to a hospital). The underlying condition may be temporary, however, and whether they limit the work capacity after discharge is not evident. It might also be so that CCS are kept for observation for, e.g., one extra day compared to references.

SA and DP capture health conditions that limit the patient’s work capacity; SA a more temporary and DP a more permanent work incapacity. Most CCS (75.3–83.2%) and references (86.7–92.6%) in our study cohort did not have any SA in SA spells >14 days nor any DP during 10 years of young adulthood. This is reassuring information for most childhood cancer patients, their parents, and healthcare providers. Nevertheless, CCS had more SADP than references, and women had more SADP than men. Survivors of CNS tumors stood out with two to three times more SADP days/person each year of follow-up and a higher risk of >90 SADP days the last year of follow-up, compared to survivors of hematological and non-CNS solid malignancies. In the analyses with only people with at least some SADP the respective year in the numerator, the mean number of SADP per year was high among CCS: 200–280 days/person/year, indicating that many of them had been on SADP for at least half-time during the respective year. Most SADP days were from DP. The most common disease categories behind healthcare use and SADP among CCS were neoplasms and psychiatric. In addition, a considerable proportion of CNS tumor survivors had healthcare and SADP due to endocrine and nervous system disorders. These observations indicate long-term or permanent work incapacity in a minority of CCS, in particular among CNS tumor survivors. Our results are in line with previous studies showing higher risk of social security benefit uptake among CCS, in particular CNS tumor survivors, compared to references [15–18]. In a Finnish study, the proportion with early retirement among survivors of CNS tumors, hematological malignancies and solid tumors were 19.7%, 6.1%, and 4.1%, respectively, and among references 1.7–2% [15]. In a Swedish study, 13.8% of CCS and 2.7% of references had at least one of four combined disability indicators (sickness pension, handicap allowance, living in the household of the parents, disability assistance) [16]. In an American study, the proportion currently enrolled in supplemental security income or social security disability insurance programs were 7.3% and 6.4% among CCS, respectively, and 2.5% and 4.8% among references, respectively [18]. In a Norwegian study, 8.4% of CCS and 2.2% of references had DP [17]. In the same study, CCS had a higher risk than references that the diagnosis associated with the attendance benefit or DP was a neoplasm, endocrine, psychiatric, nervous system, or musculoskeletal disorder [17].

We were able to analyst the outcomes stratified by sex and among both CCS and references, SADP was higher among women compared to men. This was not surprising, in Sweden as in most western countries, with high female employment frequency, SADP is higher among women than among men [36–38].

### Strength and limitations

Major strengths were the use of microdata linked from several population-based registers of high quality [20, 21, 23, 26, 34], allowing for the identification of virtually all CCSs that fulfilled the study inclusion criteria and the longitudinal design, covering ten years of young adulthood. Through the high completeness of the registers, both selection bias and loss of follow-up were avoided. Further, the use of administrative data eliminated report bias through self-reports. Therefore, the results can be considered representative for Sweden and countries with similar social insurance and healthcare systems. Another strength was the comprehensive picture of morbidity and SADP among young adult CCS and their references, achieved by combining data on inpatient care, specialized outpatient visits, and SADP in the same study population. Our study depicts secondary healthcare use, therefore, focusing on more severe morbidity. There is no nationwide register of primary healthcare in Sweden thus such healthcare visits could not be included which can be seen as both a strength and a limitation. The same can be said about not having information about shorter SA spells (≤14 days). These limitations concerned both the CCS and the references equally. Another limitation is the lack of clinical information with potential impact on future health among CCS, including cancer stage, relapse, and the treatments provided. Also, although we stratified CCS by the three main subgroups of cancer, each subgroup is still heterogeneous, including different cancer diseases and treatment protocols. This study serves as an explorative investigation in this field and future studies with good quality data are needed to identify those with the highest risk of adverse health outcome during young adulthood and also to identify different patterns of SADP during these years.

## Conclusion

Although a majority of CCS had specialized outpatient visits due to different diagnosis, only a minority had SADP during the ten-year of follow-up. Hence, having outpatient visits and diagnoses does not necessarily imply need of SA or DP. Nevertheless, CCS are at higher risk of SADP compared to references, in particular survivors of CNS tumors. Among CCS, women, those with elementary schooling, and survivors of CNS tumors are at particularly high risk of SADP and might benefit the most from preventive measures from society to help them enter and stay in the work force.

## Supporting information

S1 File (PDF)
