# Supplementary material for: Long-term diagnosis-specific sickness absence, disability pension, and healthcare use in 1305 young adult childhood cancer survivors and in 6430 references; a Swedish ten-year prospective cohort study
Source: PLoS One. 2022 Sep 30;17(9):e0275343. doi: 10.1371/journal.pone.0275343 (PMC9524675; doi:10.1371/journal.pone.0275343)

Supplementary results

Long-term diagnosis-specific sickness absence, disability pension, and healthcare use in 1305 young adult childhood cancer survivors and in 6430 references; a Swedish ten-year prospective cohort study

Authors:

Fredrik Baecklund MD PhD[1,2,3]; Kristina Alexanderson PhD[3]; Lingjing Chen MD MPH PhD[3]

Author affiliation:

[1] Department of Microbiology, Tumor and Cell Biology, Karolinska Institutet, Stockholm, Sweden

[2] Pediatric Oncology Unit, Karolinska University Hospital, Stockholm, Sweden

[3] Division of Insurance Medicine, Department of Clinical Neuroscience, Karolinska Institutet, SE-171 77 Stockholm, Sweden

Short title:

Sick leave and healthcare use after childhood cancer

S1 Table. The number (n) of childhood cancer survivors and references at the start of each of the ten follow-up years, excluding those who emigrated or died each of the ten follow-up years.

Year	Childhood cancer survivors<br>n	References<br>n
**2009**	1195	6047
**2010**	1185	6021
**2011**	1177	5980
**2012**	1171	5946
**2013**	1159	5918
**2014**	1155	5888
**2015**	1143	5856
**2016**	1137	5833
**2017**	1127	5811
**2018**	1121	5784

S1 Fig. The annual mean number of specialized outpatient visits, by main diagnoses, in the years 2009 through 2018 among young adult childhood cancer survivors and their matched references (A), stratified by sex (B), and by sex and main types of childhood cancer (C), respectively.

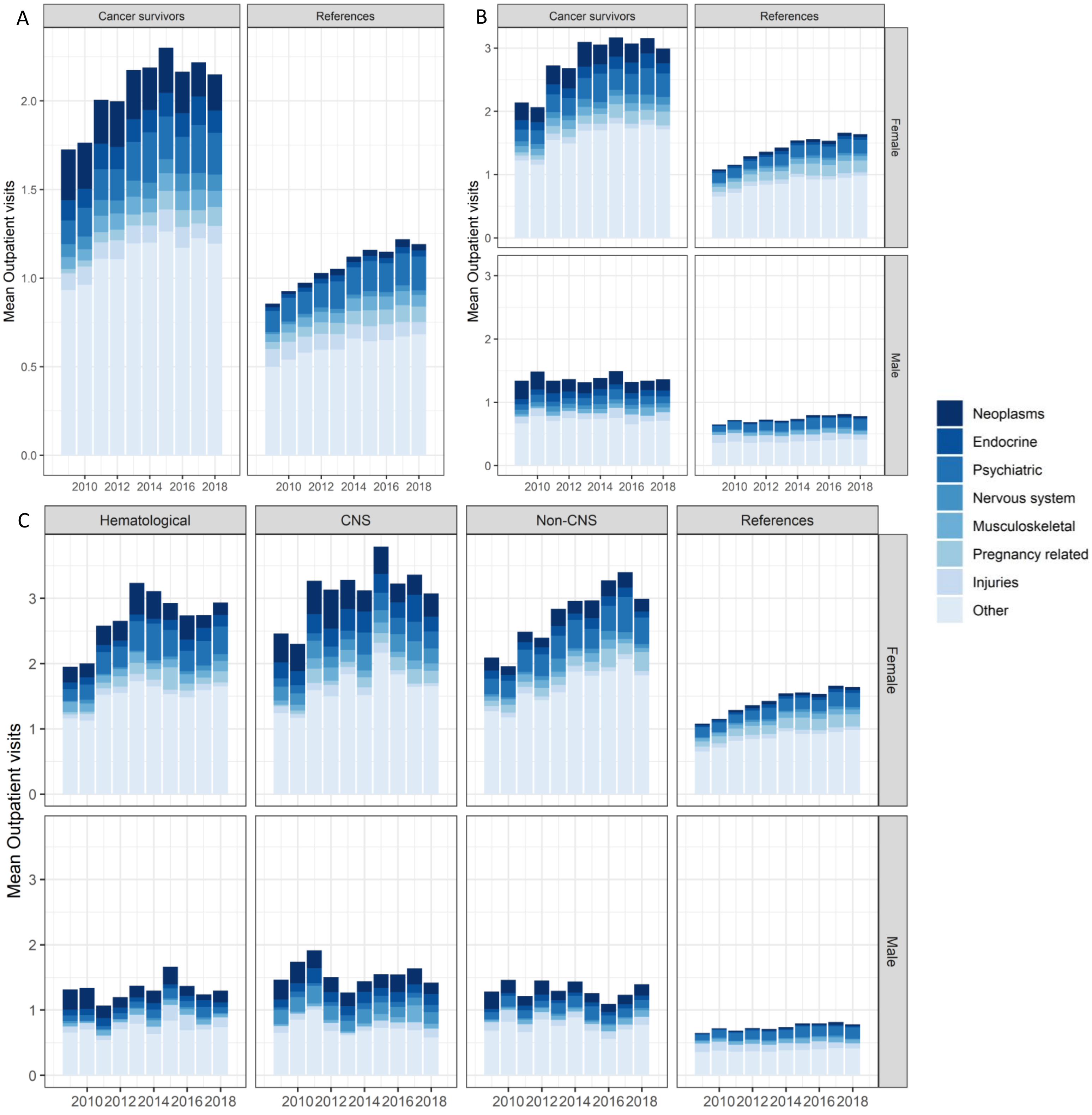

S2 Fig. The annual mean number of inpatient days by main diagnoses in each of the years 2009 through 2018 among young adult childhood cancer survivors and matched references (A), stratified by sex (B), and by sex and main types of childhood cancer (C), respectively.

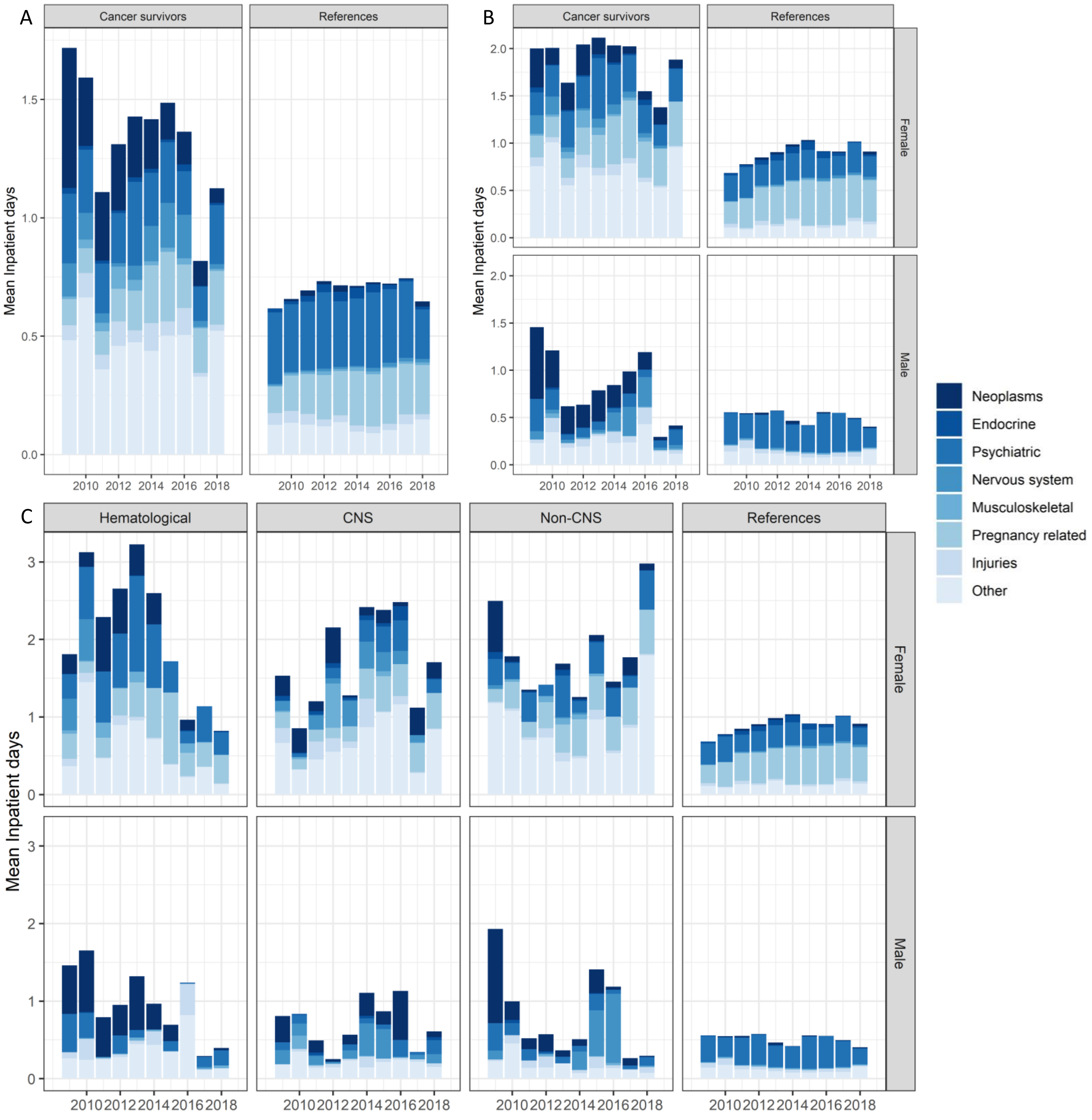

S3 Fig. The annual mean number of sickness absence (SA) and disability pension (DP) net days, by SA and DP diagnoses in each of the years 2009 through 2018 among young adult childhood cancer survivors and matched references who had any SA in spells >14 days and/or DP in the respective year (A), stratified by sex (B), and by sex and main types of childhood cancer (C), respectively.

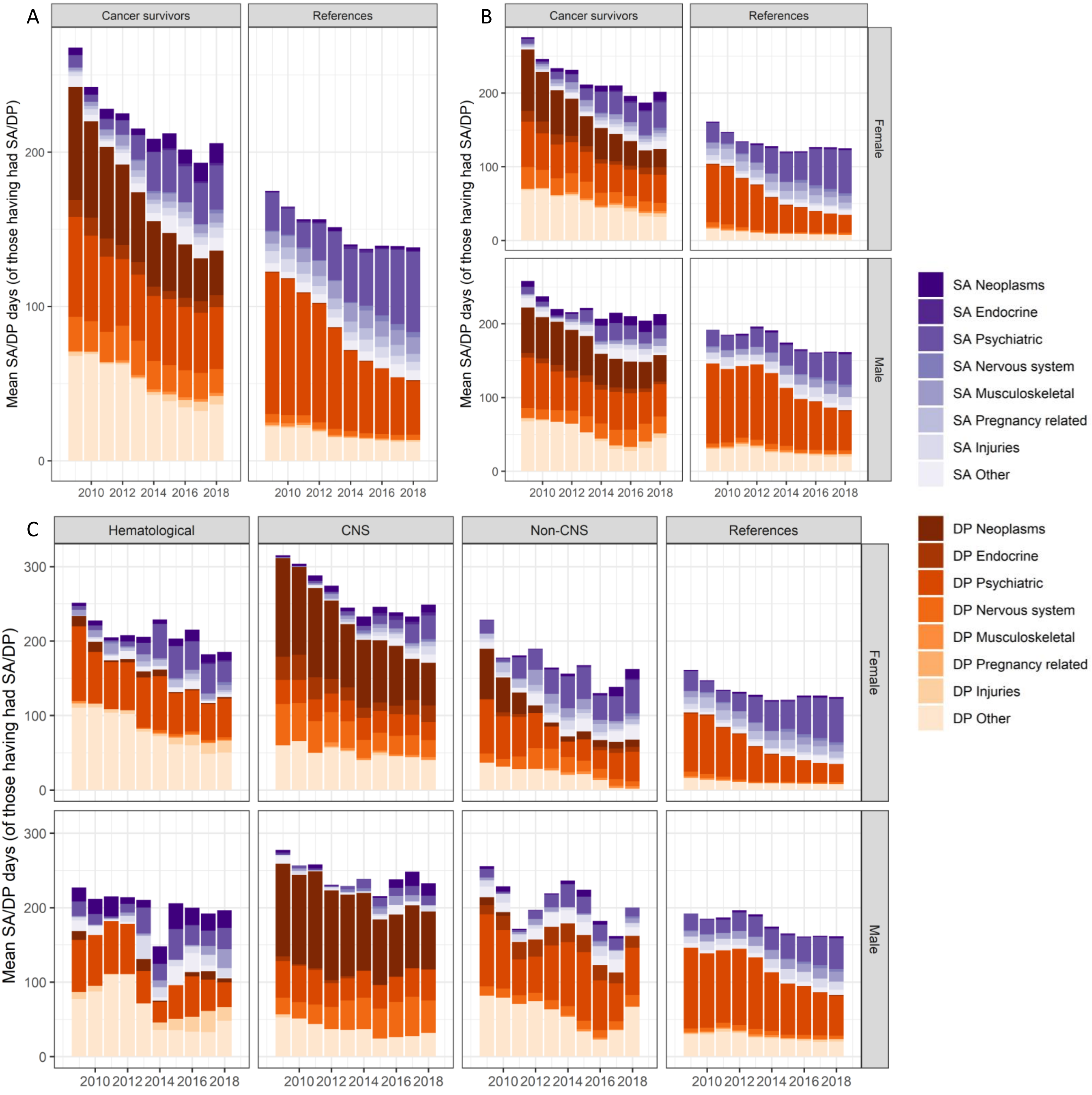

Supplement: S1 File — (PDF) [file pone.0275343.s001.pdf]
